# Blood-based biomarkers for early frailty are sex-specific: validation of a combined *in silico* prediction and data-driven approach

**DOI:** 10.1007/s11357-024-01449-w

**Published:** 2024-12-03

**Authors:** Jelle C. B. C. de Jong, Martien P. M. Caspers, Remon Dulos, Jessica Snabel, Marjanne D. van der Hoek, Feike R. van der Leij, Robert Kleemann, Jaap Keijer, Arie G. Nieuwenhuizen, Anita M. van den Hoek, Lars Verschuren

**Affiliations:** 1 Department of Metabolic Health Research, The Netherlands Organization for Applied Scientific Research (TNO), Leiden, The Netherlands; 2 Human and Animal Physiology, Wageningen University, Wageningen, The Netherlands; 3 Department of Microbiology and Systems Biology, The Netherlands Organization for Applied Scientific Research (TNO), Leiden, The Netherlands; 4 Applied Research Centre Food and Dairy, Van Hall Larenstein University of Applied Sciences, Leeuwarden, The Netherlands; 5 MCL Academy, Medical Centre Leeuwarden, Leeuwarden, The Netherlands; 6 RIC-AFL Inholland University of Applied Sciences, Delft and Amsterdam, The Netherlands

**Keywords:** Biomarker identification, Prevention, Screening, Monitoring, Diagnosis, Circulating markers

## Abstract

Frailty is characterized by loss of physical function and is preferably diagnosed at an early stage (e.g., during pre-frailty). Unfortunately, sensitive tools that can aid early detection are lacking. Blood-based biomarkers, reflecting pathophysiological adaptations before physical symptoms become apparent, could be such tools. We identified candidate biomarkers using a mechanism-based computational approach which integrates a priori defined database-derived clinical biomarkers and skeletal muscle transcriptome data. Identified candidate biomarkers were used as input for a sex-specific correlation analysis, using individual gene expression data from female (*n* = 24) and male (*n* = 28) older adults (all 75 + years, ranging from fit to pre-frail) and three frailty-related physical parameters. Male and female groups were matched based on age, BMI, and Fried frailty index. The best correlating candidate biomarkers were evaluated, and selected biomarkers were measured in serum. In females, myostatin and galectin-1 and, in males, cathepsin B and thrombospondin-4 serum levels were significantly different between the physically weakest and fittest participants (all *p* < 0.05). Logistic regression confirmed the added value of these biomarkers in conjunction with age and BMI to predict whether the subjects belonged to the weaker or fittest group (AUC = 0.80 in females and AUC = 0.83 in males). In conclusion, both *in silico* and *in vivo* analyses revealed the sex-specificity of candidate biomarkers, and we identified a selection of potential biomarkers which could be used in a biomarker panel for early detection of frailty. Further investigation is needed to confirm these leads for early detection of frailty.

**Supplementary Information:**

The online version contains supplementary material available at 10.1007/s11357-024-01449-w.

## Introduction

Frailty is characterized by loss of physical function which greatly decreases quality of life and is associated with multiple comorbidities and mortality in the elderly population [1–5]. Frailty-related physical weakness may be treated by a combination of exercise and protein supplementation, which should preferably be implemented as early as possible to prevent deterioration of physical function and disability [6]. However, user-friendly tools that can aid general practitioners to effectively diagnose frailty are lacking.

To address this problem, the SARC-F questionnaire was developed and recommended to general practitioners to use as a screening tool for sarcopenia [7, 8]. However, research also showed that the SARC-F questionnaire has a low to moderate sensitivity, meaning that it has difficulties with picking up the non-severe cases that are in the early phase of frailty, i.e., pre-frailty [9]. Novel sensitive tools for early detection would therefore be most helpful, especially since the treatment of frailty-related physical weakness would benefit from an early detection [10].

Blood-based biomarkers could be excellent candidates for early-detection of frailty-related physical weakness, as they could reflect pathophysiological adaptations before physical symptoms become apparent. Besides, they are accessible, cost-effective, and easily implementable in a variety of clinical settings [8]. Previous studies have investigated associations between frailty and potential blood-based biomarkers, which often included biomarkers related to inflammation, hematological profile or cellular senescence [11–15]. Although these biomarkers indeed reveal valuable information and can reflect the effects of aging on muscle tissue, they are largely influenced by (aging-induced) disturbances in other organs as well. It would therefore be interesting to explore potential biomarkers that are muscle tissue specific.

Importantly, research has shown that aging and frailty-associated alterations of both muscle tissue and the blood proteome contain sex-specific features [16–20]. Specifically in frailty sex differences are evident, hallmarked by female-specific associations with inflammation in muscle tissue [17] and the circulation [19, 20]. These findings highlight a potential role of sex in biomarker discovery for frailty. Therefore, we performed our analyses for each sex separately, allowing us to test whether shared or sex-specific biomarkers would be identified.

To discover novel biomarkers, we used a mechanism-based *in silico* prediction approach to identify potential muscle-secreted biomarkers [21–23]. Using a clinical biomarker database, we then evaluated whether the encoded proteins were found in circulation, endowed with a possible biomarker function and associated with potentially relevant frailty-related disease processes. Finally, the most promising biomarkers were identified using skeletal muscle transcriptome data belonging to fit and pre-frail older adults, depending on Fried criteria [24]. These were ultimately measured in serum, and their predictive value for physical function and sex-specificity were assessed.

## Materials and methods

### Study population

Functional data, muscle biopsies, and serum samples were obtained during the FITAAL study [25]. Briefly, fit and pre-frail older adults (75 + years of age) and young adults (20–30 years of age) of both sexes were recruited. For the identification of early biomarkers for frailty, data and samples of only fit and pre-frail old participants were used. In addition, to assess whether serum concentrations of these biomarkers are also different in old vs. young groups, serum concentrations were measured in serum samples of young groups as well. Male and female participants were matched in both young and old groups with respect to age and body mass index (BMI). Males and females were also matched for the Fried frailty score in the old groups. The exclusion criteria included diagnosis with cardiac failure, chronic obstructive pulmonary disease, anemia, cancer, neuromuscular disorder or dementia, contraindications for muscle biopsy, recent (up to 3 months prior to initiation of the study) significant medical or surgical events or treatment by a medical specialist, current enrolment in another study, intake of carnitine supplements, or usage of several types of medication (e.g., corticosteroids or fibrates). A BMI < 20 kg/m^2^ or > 25 kg/m^2^, diagnosis with diabetes mellitus types I and II, and a high frequency of physical exercise (> 4 times a week) served as additional exclusion criteria for young individuals, as did pregnancy or nursing for young female participants. The study was conducted according to the Declaration of Helsinki, was approved by the medical ethical committee of Wageningen University (METC nr. 16/20), and is registered in the Dutch Trial Register (NTR6124). All participants provided written informed consent prior to enrolment. An overview of subject characteristics is given in Table 1.

**Table 1:** Characteristics of all participants

	Old		Young	
	Females (*n* = 24)	Males (*n* = 28)	Males (*n* = 13)	Females (*n* = 13)
Age (years)	79.9 ± 0.6	79.7 ± 0.7	22.6 ± 0.5	23.3 ± 0.5
Weight (kg)	68.5 ± 2.2^a^	81.0 ± 2.0^b^	63.9 ± 1.7^a^	76.2 ± 2.5^b^
BMI (kg/m^2^)	26.0 ± 0.6	26.4 ± 0.7	22.2 ± 0.5	22.5 ± 0.3
400 m walk test (s)	351.7 ± 11.3^a^	323.3 ± 7.6^b^		
Time five chair stands (s)	14.3 ± 0.9	13.3 ± 0.7		
Time gait speed test (s)	4.2 ± 0.2	3.9 ± 0.2		
Fried frailty score	0.5 ± 0.1	0.5 ± 0.1		
Pre-frail (%)	41.7%	46.4%		

Superscript letters a and b denote the presence of significant difference amongst the male vs. female group. Values are averages ± SEM

### Physical function assessment

Physical function of the lower body was measured using three different tests, i.e., the 400-m walk time test, the 4-m gait speed test, and the five-time chair stand test. The tests were performed as validated previously [26, 27]. For the 400-m walk time test, a time limit of 900 s was used, which was given as a final score if participants were unable to finish the 400-m walk test. For the gait speed test, the time to walk four meters was measured. For the five-time chair stand test, participants were seated on a chair with arms folded across their chest. The time required to finish the fifth stand was used.

### Muscle biopsy and RNA-sequencing

Muscle tissue was collected by a trained physician at Leeuwarden Medical Centre and taken by percutaneous needle biopsy (50–80 mg) from the vastus lateralis muscle according to the Bergström method with suction [28, 29]. Samples were taken after an overnight fast, under local anesthesia and taken at the thickest part of the muscle, approximately 15 to 20 cm above the edge of the patella. Immediately after collection, the muscle tissue was snap frozen by immersion in liquid nitrogen and stored at − 80 °C. Total RNA was extracted from the muscle biopsies using RNA isolation kit with NucleoSpin columns (kit#740955, Macherey–Nagel). Total RNA concentration was determined spectrophotometrically using Nanodrop 1000 (Isogen Life Science, De Meern, The Netherlands), and RNA quality was assessed using the 2100 Bioanalyzer (Agilent Technologies, Amstelveen, The Netherlands). The NEBNext Ultra Directional RNA Library Prep Kit for Illumina was used to process the samples according to the protocol “NEBNext Ultra Directional RNA Library Prep Kit for Illumina” (NEB #E7420S/L). Strand-specific messenger RNA sequencing libraries were generated and sequenced at GenomeScan (Leiden, The Netherlands). The libraries were multiplexed, clustered, and sequenced on an Illumina NextSeq500 with a single-read 75-cycle sequencing protocol, 15 million reads per sample. Sequence reads were quality trimmed and mapped to the reference genome Homo sapiens GRCh38 using the trimmomatic and STAR-aligner software (GitHub). Read counts per gene transcript (counts/feature) were obtained from htseq-count software (GitHub). The gene expression dataset can be accessed from the Gene Expression Omnibus (GEO) with accession number GSE144304.

### Integrated *in silico *and data-driven approach to identify candidate biomarkers

**Fig. 1:**
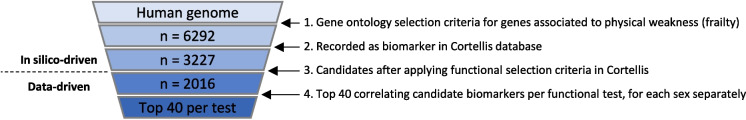
A schematic overview of the *in silico* approach for the identification of candidate biomarkers. In the first step, pathways in Gene Ontology were selected that are related to physical weakness in older adults (frailty), containing 6292 genes. Hereafter, it was tested whether these 6292 genes are recorded as biomarkers in the Cortellis database, resulting in 3227 candidate biomarkers. Additional functional selection criteria were applied in the Cortellis database (as described in the “Materials and methods” section), after which 2016 candidate biomarkers were left. Lastly, correlation analysis was performed for these 2016 candidate biomarkers between RNA-seq-derived gene expression levels and outcomes from physical function tests related to frailty. The top 40 correlating candidate biomarkers per functional test for each sex were selected for further examination

To identify candidate biomarkers, the following workflow was used (Fig. 1). Biological processes relevant for frailty were selected using gene ontology (GO) terms. Genes from these pathways were downloaded from the GO database under the conditions of species “homo sapiens” and type “protein.” Briefly, 53 GO terms related to anabolic resistance, protein synthesis, denervation, endocrine alterations, fat infiltration, inflammation, differentiation, myofiber type switching, cellular senescence, oxidative stress, physical activity, mitochondrial dysfunction, transmembrane transport, and cell death were selected (a complete list of the selected GO terms is added as Supplementary Table 1). This resulted in an initial set of 6292 genes (Fig. 1). Next, we downloaded information on these protein-encoding genes in regard to their potential role as blood-based biomarker from the Clarivate Analytics Integrity biomarker database (accessed 2021) was used to see. This database contains an array of information about whether proteins have previously been described as a biomarker in literature, as well as additional filter options, e.g., in regard to the type of study they have been measured in. In the Clarivate database 3227 of the selected protein-encoded genes were found to be previously described as a biomarker. Additional filter options for population (“All” or “Adult”), type (“Serum” or “Plasma”), Validity (“Early studies in humans,” “Late studies in humans,” or “Recommended/Approved”), Condition (“Sarcopenia,” “Muscle wasting,” “Muscular atrophy,” “Generalized weakness,” “Oxidative stress,” “Metabolic syndrome,” “Insulin resistance,” “Muscle disorders,” “Nerve and muscle disorders,” “Calcium metabolism disorders,” “Vitamin D deficiency” or “Inborn errors of metabolism”) and role (“(Differential) Diagnosis,” “Disease profiling,” “Monitoring disease progression,” “Monitoring treatment efficacy,” “Predicting treatment efficacy,” “Prognosis (– risk stratification),” “Risk factor,” or “Selection for therapy”) were used as well in the Clarivate database and this resulted in a selection of 2016 candidate biomarkers.

Using this selection of candidate biomarkers for frailty, the workflow continued using a data-driven approach. Normalized RNA-seq-derived counts of reads mapped on the genes encoding the 2016 selected candidate-biomarkers were used for correlation analysis, in which the correlation with the frailty-related physical function parameters (400-m walk time, five chair stands and 4-m gait speed) was tested. Both Pearson and Spearman correlations were calculated, and candidate biomarkers were ranked according to the corresponding *R*-values. If the direction of the correlation (positive or negative) was different in the Pearson and Spearman correlation test (e.g., one test indicating a negative correlation and the other a positive correlation), then the value of 0 was assigned. A dedicated evaluation of the top 40 correlating genes was made for each physical function test, based on their relation to the physical fitness parameters, the existence of an active secretion mechanism or their release due to tissue damage, muscle-specificity of the protein, and availability of appropriate ELISA kits.

### Blood collection and ELISA

Blood samples were taken from participants in the morning after an overnight fast and collected using serum separating tubes (BD diagnostics). Serum samples were stored at − 80 °C until analysis. To test our shortlist of candidate biomarkers, cathepsin B (R&D systems, DY2176), thrombospondin-4 (Thermo Fisher, EH473RB), galectin-1 (R&D systems, DY1152-05), myostatin (R&D systems, DY788-05), and titin-N-fragment (IBL, 27,902) serum concentrations were determined using commercial ELISA kits. Quality of ELISAs was assessed by performing spike recovery tests and serial dilutions (data provided in Suppl. Table 2). Assays were performed according to manufacturer instructions. In case of myostatin, samples were activated to measure the concentration of total myostatin using a sample activation kit (R&D systems, DY010).

### Statistical analysis

For the statistical analysis of biomarker serum concentration data, participants were grouped into tertiles for each sex separately based on their performance during the physical function tests. Comparisons were made with the best performing tertile (T1) for each sex separately. For this purpose, the first normality of data was tested using a Shapiro–Wilk normality test. If data was normally distributed, then statistical differences between the first tertile and other tertiles were tested using a one-way ANOVA, followed by a Dunnett’s test for post-hoc analysis (1-sided). If data was not normally distributed, then groups were compared using a Kruskal–Wallis test followed by a Mann–Whitney U test (1-sided). If data belonging to only two groups were compared (e.g., old vs. young participants), then an independent *t*-test was used, or a Mann–Whitney *U* test in case of not normally distributed data. Logistic regression analysis was performed to assess the predictive capacity (sensitivity and specificity) of different variables for physical function status, which was used as input for receiver-operator-curve (ROC) and to quantify the area under the curve (AUC). A *p*-value of < 0.05 was considered statistically significant, and all values are displayed as mean ± SEM. Statistical analyses were performed using IBM SPSS Statistics 27 (IBM, NY, USA).

## Results

### Top 40 candidate biomarkers differ per physical test, but overall respond similarly

**Fig. 2:**
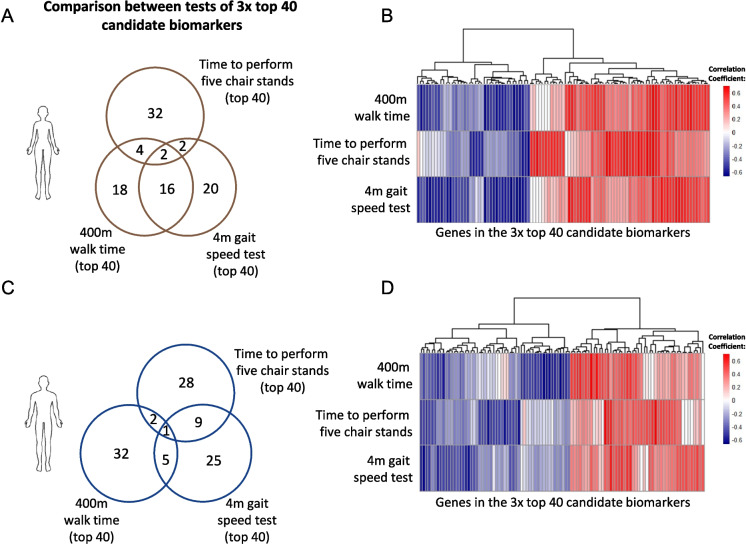
Visual representation of overlap in top correlating candidate biomarkers between the three physical function parameters. **A** Venn diagram indicating the number of shared or unique candidate biomarkers for each physical function outcome in females. **B** Heatmap with hierarchical clustering displaying the correlation coefficient for each candidate biomarker across each physical function parameter in females. A red color indicates a positive, and a blue color indicates a negative correlation between the RNA-seq-derived expression levels of a gene and the respective physical function parameter. **C** Venn diagram indicating the number of shared or unique candidate biomarkers for each physical function parameter in males. **D** Heatmap with hierarchical clustering displaying the correlation coefficient for each candidate biomarker across each physical function parameter in males

Potential biomarkers were selected *in silico* using transcriptome data of the *vastus lateralis* muscle from fit and older adult prefrail individuals using the workflow described in Fig. 1. Correlation coefficients were calculated for the 2016 *in silico* predicted candidate biomarkers. The correlation coefficients of these candidate biomarkers were calculated based on the correlation between RNA-seq-derived gene expression in vastus lateralis muscle tissue, and three frailty-related physical function tests, i.e., the 400-m walk time test, five-chair stand test, and 4-m gait speed test. The top 40 correlating candidate biomarkers from each physical function test (correlations ranging from an absolute *r* of 0.43 to 0.75) were examined for overlap between the three physical function tests (Fig. 2A). In females, a total of 94 unique candidate biomarkers were identified. Notably, the top 40 candidate biomarkers were mostly specific for each physical test. Some overlap (*n* = 16) was found between the 400-m walk time and 4-m gait speed test. A heatmap using the correlation coefficients of the 94 unique candidate biomarkers with all three physical function tests revealed that candidate biomarkers mostly tended to correlate in a similar direction (positive or negative) for all three physical function tests (Fig. 2B).

In the males a total of 102 unique candidate biomarkers were identified, and also these were mostly specific for one of the three top 40 candidate biomarkers (Fig. 2C). Only one biomarker was shared by all three physical function tests. Similarly as found in females, these candidate biomarkers did tend to correlate in similar direction for all three physical function tests (Fig. 2D).

### *In silico *workflow predicts candidate biomarkers to be sex-specific

**Fig. 3:**
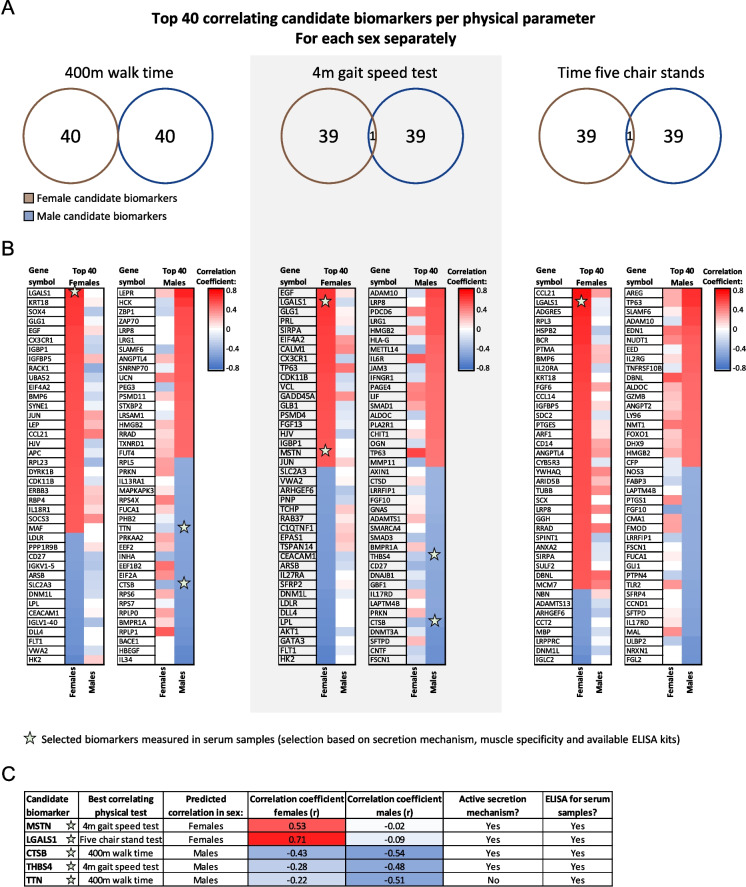
Visual representation of overlap in top correlating candidate biomarkers between female and male older adults. **A** Venn diagram indicating the number of shared or unique candidate biomarkers between the two sexes for each physical function parameter. **B** Heatmap displaying pairwise (for both females and males) the correlation coefficient of the top 40 candidate biomarkers for each physical function test. Stars indicate that the candidate biomarker was selected for measurement in serum using ELISA. A red color indicates a positive, and a blue color indicates a negative correlation between the RNA-seq-derived expression data of a gene and the respective physical function parameter. **C** A table highlighting the selected candidate biomarkers for measurement in serum using ELISA

Venn diagrams were created to visualize the overlap between the two sexes with respect to the top 40 candidate biomarkers for each physical function test (Fig. 3A). Strikingly, hardly any overlap was found between the female and male top 40 candidate biomarkers, indicating highly sex-specific correlations between the RNA-seq data and the frailty-related physical function tests. To examine similarity in direction of correlation, correlation coefficients of both sexes were visualized alongside each other for each of the top 40 candidate biomarkers for both sexes (Fig. 3B). This revealed that similarity in direction of correlation was frequently not the case (in 49.6% of the biomarkers). For example, some genes (e.g., LGALS1, IGBP1, and PRKN) did not exhibit a consistent negative or positive correlation with a physical function parameter across the two sexes.

In the next step of the workflow, candidate biomarkers were selected to be measured in serum samples for validation. Several criteria were assessed, such as the existence of an active secretion mechanism or its release due to tissue damage, muscle-specificity of the protein, and availability of appropriate ELISA kits (Fig. 3C). Based on these criteria, myostatin (MSTN), galectin-1 (LGALS1), cathepsin B (CTSB), thrombospondin-4 (THBS4), and titin (TTN) were selected and measured in serum samples. MSTN (*r* = 0.53 with 4-m gait speed test) and LGALS1 (*r* = 0.71 with five chair stand test) were predicted to correlate with physical function in the female older adults. CSTB (*r* =  − 0.54 with 400-m walk time), THBS4 (*r* =  − 0.48 with 4-m gait speed test), and TTN (*r* =  − 0.51 with 400-m walk time) were predicted to correlate with physical function in the male older adults.

### Myostatin and galectin-1 correlate with physical weakness in female, but not in male older adults

**Fig. 4:**
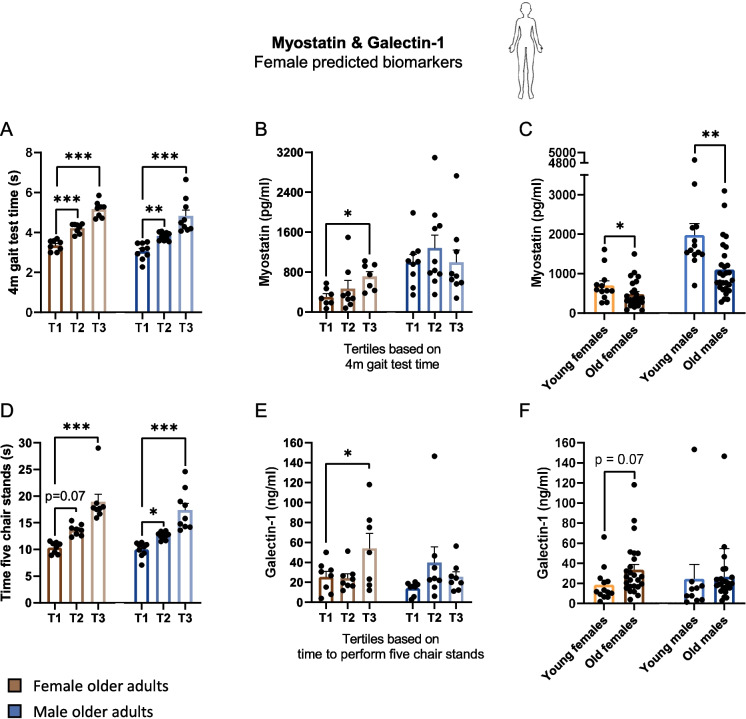
Changes in myostatin and galectin-1 serum concentrations during frailty and aging. **A** Female (brown) and male (blue) tertiles as defined based on their 4 m gait test time. **B** Myostatin serum concentration levels in the female and male tertiles. **C** Myostatin serum concentration levels in young and old groups. **D** Female and male tertiles as defined based on their time to perform five chair stands. **E** Galectin-1 serum concentration levels in the female and male tertiles. **F** Galectin-1 serum concentration levels in young and old groups. Values represent mean ± SEM, *p < 0.05, **p < 0.01, and ***p < 0.001

The older adults were divided into tertiles based on their 4-m gait speed test time, since myostatin serum levels were predicted to correlate with this parameter in female older adults (*r* = 0.53, Fig. 3C). Compared to the first tertile, older adults in the second (*p* < 0.01) and third tertile (*p* < 0.001) of both sexes were significantly slower during their 4-m gait speed test time (Fig. 4A). In females, serum levels of total myostatin were higher in the third tertile compared to the first tertile (*p* < 0.05), while in males no significant differences were found between the tertiles (Fig. 4B). Besides differences within the groups of older adults, we were interested in testing whether differences in serum levels of old and young participants could be detected. This was performed to test whether the differences in the frailty-related physical weakness readout parameters are a continuum of aging. Strikingly, in both the male and female groups, a significantly lower myostatin serum level was measured in the old groups compared to the young groups (*p* < 0.05 in females and *p* < 0.01 in males, Fig. 4C). This indicates a different correlation between the serum concentration of myostatin and (1) age and (2) pre-frailty (going down during aging, but up during pre-frailty).

Galectin-1 was predicted to correlate best with time to perform five chair stands in females (*r* = 0.71, Fig. 3C), and the older adults were separated into tertiles based on time to perform five chair stands (Fig. 4D). Female older adults in the second tertile tended to be significantly slower compared to female older adults in the first tertile (*p* = 0.07), and females in the third tertile were significantly slower compared to females in the first tertile (*p* < 0.001). Males in the second and third tertile were significantly faster than males in the first tertile (*p* < 0.05 and *p* < 0.001, respectively). In females, a significantly higher serum concentration of galectin-1 was measured in the third tertile compared to the first tertile (*p* < 0.05, Fig. 4E). In males, no significant differences were found across the tertiles. Consistently, in old compared to young females, a tendency for a significant increase in galectin-1 serum levels was found (*p* = 0.07), but no such tendencies were found in the serum concentration in old compared to young males (Fig. 4F).

### Cathepsin B and thrombospondin-4 correlate with physical weakness in male, but not in female older adults

**Fig. 5:**
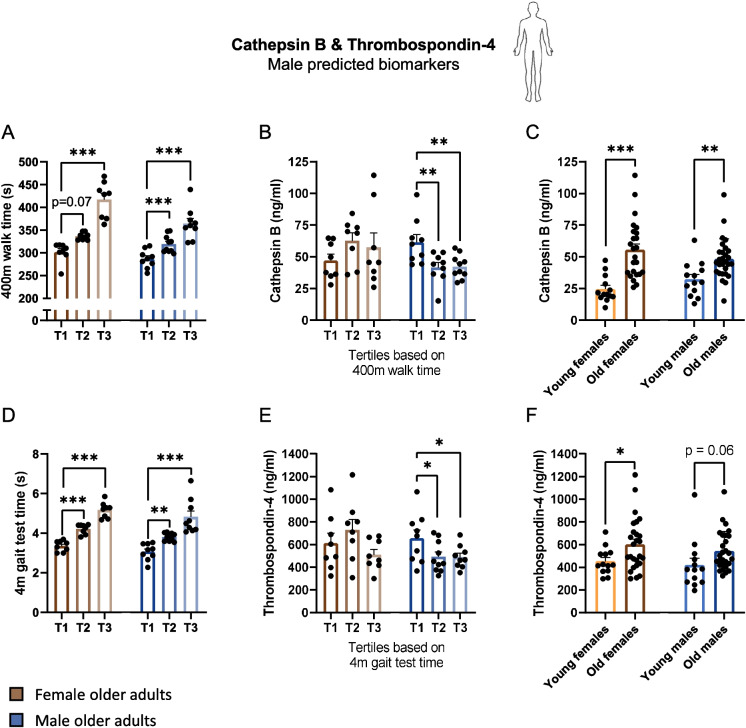
Changes in cathepsin B and thrombospondin-4 serum concentrations during frailty and aging. **A** Female (brown) and male (blue) tertiles as defined based on their time to walk 400 m. **B** Cathepsin B serum concentration levels in the female and male tertiles. **C** Cathepsin B serum concentration levels in the young and old groups. **D** Female and male tertiles as defined based on their 4 m gait test time. **E** Thrombospondin-4 serum concentration levels in the female and male tertiles. **F** Thrombospondin-4 serum concentration levels in the young and old groups. Values represent mean ± SEM, *p < 0.05, **p < 0.01, and ***p < 0.001

Figure 5A shows a separation of the older adults into tertiles based on their 400-m walk time (Fig. 5A), since cathepsin B serum levels were predicted to correlate best with this parameter in the male older adults (*r* =  − 0.54, Fig. 3C). Time to walk 400 m tended to be significantly higher in the second tertile of the female older adults compared to the first tertile (*p* = 0.07), and was significantly higher in the third tertile of the female older adults compared to the first tertile (*p* < 0.001). Time to walk 400 m was significantly higher in both the second and third tertile of the male older adults compared to the first tertile (*p* < 0.001). Serum concentrations of cathepsin B were not different among the tertiles in females but were significantly lower in the second and third tertile of the male older adults (*p* < 0.001, Fig. 5B). Contrastingly, serum concentrations of cathepsin B were higher in older adults compared to the younger groups (*p* < 0.001 in females and *p* < 0.01 in males, Fig. 5C). Similarly, as myostatin, these data indicate a differential correlation between cathepsin B and (1) age and (2) pre-frailty (serum concentration going down during pre-frailty, but up during aging).

In Fig. 5D, the older adults were separated into tertiles based on their 4-m gait test time (Fig. 5D), since thrombospondin-4 serum levels were predicted to correlate best with this parameter in male older adults (*r* =  − 0.48, Fig. 3C). Times to complete the 4-m gait test were significantly higher in the second and third tertiles of both sexes compared to the first tertiles (*p* < 0.01, Fig. 5E). In female older adults, no differences were found between the tertiles in regard to the serum concentration of thrombospondin-4. In male older adults, thrombospondin-4 was significantly higher in the fastest tertile, compared to slower tertiles (*p* < 0.05, Fig. 5E). Contrastingly, serum concentrations of thrombospondin-4 were significantly higher or tended to be higher in the old groups compared to young groups (*p* < 0.05 in females and *p* = 0.06 in males, Fig. 5F).

Lastly, titin N-fragment was measured as well since it was predicted to correlate with time to walk 400 m in males (*r* =  − 0.51, Fig. 3C), although titin is not actively excreted unlike the other selected candidate biomarkers. No differences in serum concentration of titin N-fragment between the tertiles were observed in both sexes (Suppl. Figure 1). Interestingly, titin N-fragment concentration was higher in the older vs. young groups of both sexes (*p* < 0.01, Suppl. Figure 1). Furthermore, differences between tertiles were tested as well if tertiles were based on physical function data other than the physical function parameter predicted to correlate with these biomarkers. No significant differences between these tertiles were found (Suppl. Figure 2), indicating test-specific correlations between biomarkers and tests of physical function. Lastly, correlations between serum concentrations of the biomarkers were calculated as well, and in females, a tendency (*p* = 0.06) for a correlation between myostatin and galectin-1 serum concentrations was found, while a significant correlation between cathepsin B and galectin-1 serum concentrations was found (Suppl. Figure 3A). In males, a significant correlation between myostatin and galectin-1 serum concentrations was found as well (Suppl. Figure 3B).

### Biomarkers are of added value compared to age and BMI to identify the weakest older adults

**Fig. 6:**
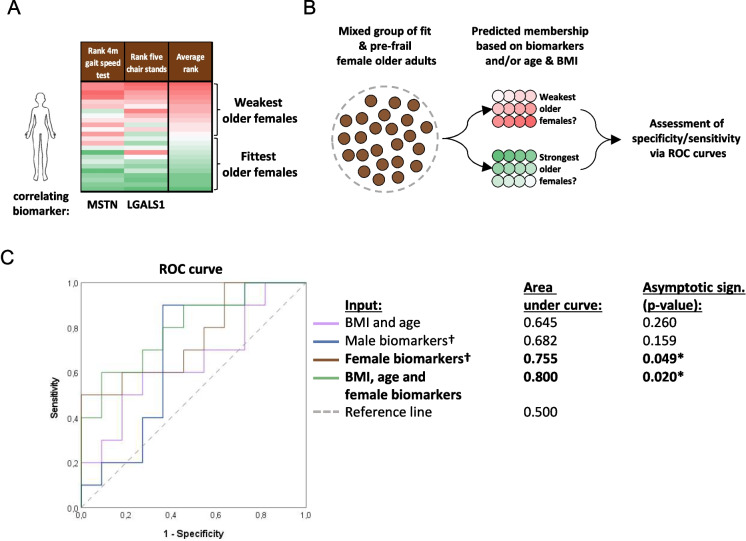
Comparison between the predictive capacity of the biomarkers, BMI and age for the membership of a female older adult towards either the fittest or weakest half of the female study population. **A** A heatmap visualizing the classification of participants towards the weakest or fittest half of the female population. Data of physical function parameters correlating with identified biomarkers were used (4-m gait speed test and time to perform five chair stands). **B** Simplified visualization of logistic regression analysis. **C** ROC curve visualizing area under the curve for the different variables used as input

We next performed logistic regression analysis to assess whether the identified biomarkers are of added value on top of age and BMI to predict physical weakness in older adults. Age and BMI are both considered to be predictors of frailty [30, 31]. The predicted outcome was whether the older adult belonged to the fittest or the weakest half of the group. Female older adults were separated into fittest and weakest half based on their performance during the 4-m gait speed test and five chair stands test (Fig. 6A), since these physical function parameters correlated with the “female” biomarkers (myostatin and galectin-1). Besides the serum concentration level of the biomarkers, other variables, such as age and BMI, were tested as well for their predictive capacity of physical function (Fig. 6B). The AUCs were determined for the identification of the fittest and the weakest female older adults (Fig. 6C). The AUCs of age and BMI, and the “male” biomarkers (cathepsin B and thrombospondin-4) were not significantly different from the AUC of the reference line, as indicated by the asymptotic sign. > 0.05. This reveals that both the age and BMI, as well as the “male” biomarkers, were not of added value for the identification of the weakest female older adults. Noticeably, the AUC of the “female” biomarkers (0.755) was significantly higher than the reference line (*p* < 0.05), indicating an added value of the female biomarkers for the identification of the weakest female older adults in this population. The AUC increased slightly to 0.8 when age & BMI were added to the female biomarkers in the model (*p* < 0.05).

**Fig. 7:**
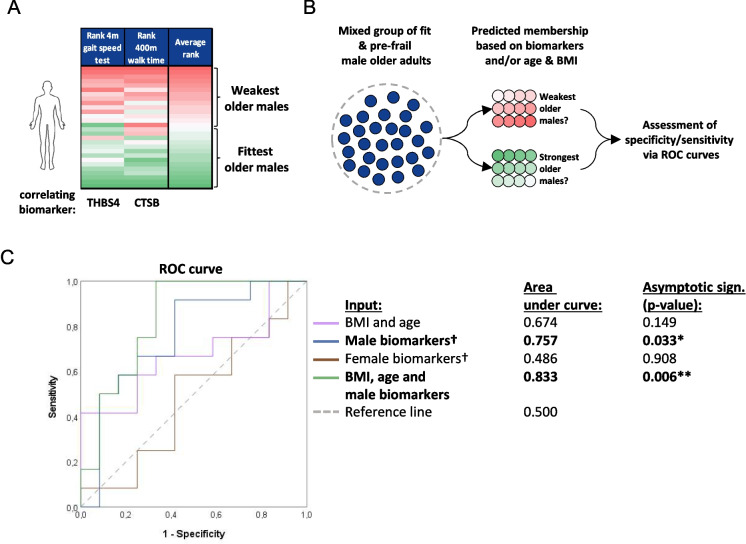
Comparison between the predictive capacity of the biomarkers and BMI and age for the membership of a male older adult towards either the fittest or weakest half of the male study population. **A** A heatmap visualizing the classification of participants towards the weakest of fittest half of the male population. Data of physical function parameters correlating with identified biomarkers were used (4-m gait speed test and 400-m walk time). **B** Simplified visualization of logistic regression analysis. **C** ROC curve visualizing area under the curve for the different variables used as input

The male older adults were separated into the weakest and fittest group based on their performance during the 4-m gait speed test and 400-m walk time test (Fig. 7A), again since these physical function parameters correlated with the ‘male’ biomarkers (cathepsin B and thrombospondin-4). Also here, different variables were used as input to predict whether each male older adults belonged to the weakest or fittest group (Fig. 7B). The AUCs of age and BMI and the “female” biomarkers were not significantly different from the reference line. The AUC of “male” biomarkers was 0.757 and was significantly higher than the AUC of the reference line (*p* < 0.05, Fig. 7C). The addition of age and BMI increased the AUC somewhat to 0.833 (*p* < 0.01).

## Discussion

The aim of this study was to identify blood-based biomarkers associated with early frailty-related physical weakness, and to investigate whether these are sex-specific. By correlating muscle transcriptome data of *in silico* selected candidate biomarkers with the physical function of fit and pre-frail elderly, we were able to identify such biomarkers, which indeed were sex-specific. In females, myostatin and galectin-1, and in males, cathepsin B and thrombospondin-4 correlated with physical weakness. Logistic regression analysis revealed the added value of these biomarkers, in conjunction with age and BMI, to predict the physical function status of the older adults. Furthermore, we showed that these biomarkers correlated differently with aging, indicating that it cannot be assumed that biomarkers for aging can be used as biomarkers for frailty-associated physical weakness as well. To our knowledge, the results of the current study are unique, since blood-based biomarkers for frailty, let alone pre-frailty, are completely lacking. Using our mechanism-based *in silico* approach we created leads for further studies in this field.

Myostatin is a well-known protein secreted specifically by muscle. It inhibits muscle mass growth and is therefore of interest with regard to its potential role as biomarker [32]. Here, we found a female-specific negative correlation between gait speed and myostatin serum concentration levels. In literature, varying results on the relation of circulating myostatin levels with frailty have been found. In our study and in the study of Wang [33] and Chew [34] et al., weaker or frail participants displayed higher serum concentrations of myostatin, while other studies found the opposite, namely a lower concentration of myostatin in serum of frail participants [35, 36]. These contrasting findings may be due to the dual role myostatin has in regard to its role as a biomarker for muscle mass and function. On the one hand, myostatin is a muscle-specific protein, implicating that a higher percentage of lean body mass could correlate with a higher serum concentration of myostatin. On the other hand, myostatin negatively regulates muscle mass growth, implicating that a higher concentration of myostatin could correlate with lower muscle mass maintenance. Thus, perhaps, myostatin has differential predictive value depending on the stage of frailty. The type of correlation observed may also be due to differences in the populations used in these studies; hospitalized older adults [35] or older adults with chronic heart failure [33]. Lastly, the outcome may also depend on whether total myostatin [35, 36] or only active myostatin was measured, but this was not always explained in mentioned publications [33, 34]. Furthermore, our findings in regard to the sex-specificity of myostatin’s role as biomarker are not in line with those of Chew et al., which found a male-specific correlation between the serum concentration of myostatin and frailty [34]. Differences here could also be explained by the age of the study population, the stage of frailty or normalization of data. In mouse studies, skeletal muscles of female mice may be more dependent on myostatin compared to those of male mice. One study, using *MSTN*^*−/−*^ mice, found that female, but not male *MSTN*^*−/−*^ mice, displayed an increase in peak-tetanic force compared to wildtype control mice [37]. Another study showed a female mice-specific reduction of myostatin to doxycycline [38]. In contrast to the human studies, these mouse studies are highly controlled, and are thereby more likely to isolate the effects of sex. However, the translatability of sex differences in muscle-aging from mice to humans should be interpreted with caution [39]. Based on the cited studies and our results, myostatin remains of interest, and its role as biomarker is likely to be sex-dependent. Longitudinal studies following subjects from fit to pre-frailty and eventually full frailty could shed light on the capacity of myostatin as a predictive biomarker for (pre-)frailty.

Galectin-1 is a lectin that stimulates the differentiation of satellite cells [40], and can thereby regulate myotube size and regeneration [41]. Galectin-1 also plays a protective role in inflammation, as it can inhibit the innate immune response [42], and has been shown to colocalize with infiltrating leukocytes in muscle tissue [43]. Since we previously observed an increased leukocyte infiltration in the weakest vs. the fittest females of this population [17], we hypothesize that an increased level of circulating galectin-1 can reflect an increased level of infiltrating leukocytes in skeletal muscle. This would also explain the female-specific correlation between galectin-1 and physical weakness, since we observed an increased expression of inflammatory markers in skeletal muscle tissue of the weakest vs. the fittest females, but not males [17]. Other studies have also suggested the existence of a female-specific role of inflammation in the development of frailty. For example, female-specific correlations between frailty and specific immune cell subpopulations [20] and inflammatory markers [44] have previously been reported. Perhaps, the origin of the females-specific upregulation of inflammation during frailty can be found in the menopause, since IL-6 and TNFα concentrations during this period [45–48]. Possibly, menopause-induced loss of estrogen plays a role in this, since estrogen can decrease TNFα secretion [49]. We now propose galectin-1 as a potential biomarker for pre-frailty related to inflammation in muscle tissue. However, its applicability in practice is yet to be proven and needs to be reproduced in other studies, since inflammation in other tissue may also increase the serum concentration of galectin-1 [50]. The latter may be problematic, since co-morbidities are highly prevalent in older adults.

Cathepsin B is a protease that is excreted after exercise, as its serum levels are increased after both acute [51] and long-term [52] exercise training. Cathepsin B is mainly known for its pro-cognitive effects, and is considered as a beneficial mediating factor between exercise and the pro-cognitive effects of exercise [53, 54]. In the current study, circulating cathepsin B correlated with 400-m walk time in male participants, but not in female participants. Based on the previously mentioned studies, we hypothesized that cathepsin B serum levels reflect physical activity, and that fit older adults are in general more physically active, and that for the same reason cathepsin B was identified as a candidate biomarker in the current study. Sex-differences in cathepsin B expression, secretion or degradation are not described in literature. Only one study found a sex-specific correlations between circulating cathepsin B levels and age and some pulmonary parameters [55]. Due to the novelty of research investigating the role of sex in cathepsin B metabolism, it is difficult to speculate about possible explanations for the male-specific correlation as found in the current study. Noticeably, in females, cathepsin B gene expression level also moderately correlated with 400-m walk time (*r* =  − 0.43, Fig. 3C). Consequently, it is likely that the lack of correlation between circulating cathepsin B protein levels and physical function in females may be explained by post-transcriptional processes, and perhaps even by processes outside of the myofibers.

Thrombospondin-4 is an extracellular protein involved in extracellular matrix assembly [56], and if mutated gives rise to muscular dystrophy [57], similar to other structural proteins such as dystrophin [58] and collagen [59]. *In vitro*, thrombospondin-4 has been shown to promote synaptogenesis, and its expression was increased after denervation [60]. The potential role of thrombospondin-4 in frailty is hardly studied, and to our knowledge, the current study is the first to suggest it as a biomarker for early frailty. Interestingly, the fittest male older adults had higher serum levels of thrombospondin-4 compared to weaker males. Possibly, thrombospondin-4 plays a protective role against the development of frailty-related physical weakness, and it is therefore a biomarker of interest for early frailty detection.

Correlations between biomarker serum concentration and physical function are shown to be dependent on the specific physical function test being employed. This is based on the observation that no statistically significant differences were detected when tertiles were based on tests other than the physical function test the biomarker was predicted to correlate best with (Suppl. Figure 2). This is not unexpected, since different tests likely utilized different mechanisms for, e.g., energy production (aerobic vs. anaerobic) and may utilize different myofiber types, containing different proteomes. Other cross-sectional research studying correlations between physical function tests and the muscle transcriptome also report differential correlations between gene expression and specific types of physical function tests (e.g. walking speed and leg power) [61, 62]. This observation suggests muscle biomarkers can reflect specific muscle functions. For example, cathepsin B, which serum concentrations increase after aerobic exercise [63], correlated with tertiles based on 400-m walk time (Fig. 5B), but not with time to perform five chair stands (Suppl. Figure 2). Consequently, it can be hypothesized cathepsin B serum concentrations are mostly affected by aerobic physical function. However, it should be taken into account this finding could also be a result of our approach for biomarker selection, which was performed for each physical function test separately. If we had used a composite score for physical function, perhaps we would have found biomarkers correlating with multiple tests for physical function.

It is commonly assumed that frailty is a continuum, or an exaggerated form of aging, and that therefore mechanisms and biomarkers of aging are the same as mechanisms and biomarkers of frailty. However, we here show that the measured blood-based biomarkers may have differential correlations to aging and frailty-related physical weakness. For example, myostatin was decreased in old vs. young female participants, whereas it was increased in the weakest vs. the fittest female older adults. Similar inconsistent differences along the aging-frailty axis were found for cathepsin B and thrombospondin-4. Only galectin-1 serum levels were increased in the old vs. young group, and also increased in the weakest vs. fittest female older adult groups (Fig. 4E, F). Overall, our findings are in line with the results of a previous publications using the same study population, in which it was shown that the intramuscular changes associated to aging are different from those associated to frailty-related physical weakness [17]. Our findings are in line as well with a recent publication describing aging related adaptive biomarkers are different from aging related damage biomarkers [64], showing a differential axis of aging related damage vs. adaptation. Together, these findings suggest biomarkers can have dual roles, which has to be taken into account while designing biomarker studies. The lack of robust frailty-specific biomarkers remains a knowledge-gap that needs to be addressed in order to ultimately identify clinically relevant blood-based biomarkers for frailty [65]. Our data also highlight the importance of matching fit and frail groups with regard to their age, since age might have a confounding effect on the serum concentrations of the biomarkers.

A limitation of the current study is the sample size, which was small since we aimed for highly matched male and female groups with regard to BMI, age, Fried frailty score, and absence of comorbidities, limiting the recruitment of a high number of participants. In addition, for the current *in silico* approach muscle biopsies are required, which are not widely available. Furthermore, we compared the predictive capacity of the identified biomarkers for physical function with age and BMI, but not with other parameters of clinical interest, such as the SARC-F questionnaire, which would have been of added value. In future research it would be of interest as well to apply multianalyte assays to measure a bigger number of analytes. Lastly, biomarkers for pre-frailty are not necessarily biomarkers for frailty per se, which should be taken into account while performing further research.

We conclude that early biomarkers for early frailty-related physical weakness are likely to be sex-specific, as found in both our *in silico* and *in vivo* analyses. In addition, we conclude that biomarkers for early frailty-related physical weakness and aging are not the same. We identified a selection of potential biomarkers (myostatin and galectin-1 in serum of females, and cathepsin B and thrombospondin-4 in serum of males), which perhaps could be used in a biomarker panel for early frailty detection; however, this needs to be replicated in other independent studies.

## Supplementary Information

Below is the link to the electronic supplementary material.

Supplementary file1; Suppl. Fig. 1. Data of serum concentration of Titin N-fragment. (A) 400m walk time data across the female and male tertiles. (B) Titin N-fragment serum concentration data in the female and male tertiles. (C) Titin N-fragment serum concentration data in the young and old groups (PDF 234 KB).

Supplementary file2; Suppl. Fig. 2. Data of serum concentrations of Myostatin (A+B), Galectin-1 (C+D), Cathepsin B (E+F) and Thrombospondin-4 (G+H) using tertiles based on alternative physical function tests, i.e. the physical function tests these biomarkers did not correlate with based on the RNA-seq data (PDF 224 KB).

Supplementary file3; Suppl. Fig. 3. Correlations between biomarker serum concentrations for (A) females and (B) males. *p 0.05 (PDF 543 KB).

Supplementary file4; Suppl. Table 1. An overview of the gene ontology terms used to identify genes involved in frailty-related physical weakness, which was the first step of the *in silico* approach to identify candidate biomarkers (XLSX 13 KB).

Supplementary file5; Suppl. Table 2. Quality control data of ELISA kits used in this study (DOCX 16 KB).

## Data Availability

The gene expression dataset can be accessed from the GEO-database with accession number GSE144304. Other data are available from the corresponding author upon reasonable request.
