# Supplementary figures and images for: Blood-based biomarkers for early frailty are sex-specific: validation of a combined in silico prediction and data-driven approach
Source: GeroScience. 2024 Dec 3;47(3):3741–58. doi: 10.1007/s11357-024-01449-w (PMC12181598; doi:10.1007/s11357-024-01449-w)

S1A

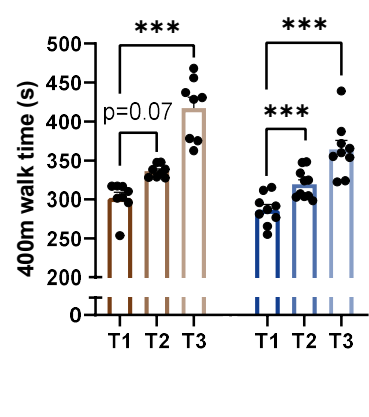

B

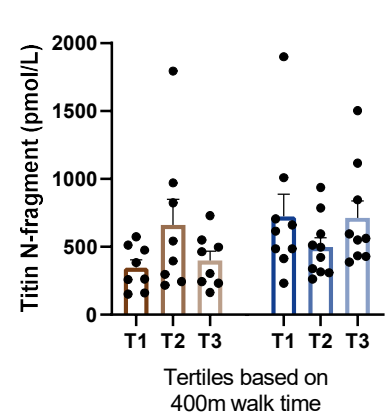

C

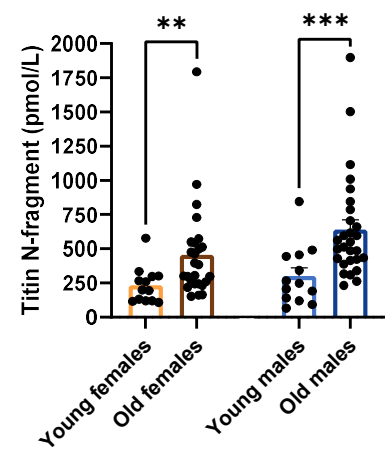

Supplement: Supplementary file 1 — Supplementary file1; Suppl. Fig. 1. Data of serum concentration of Titin N-fragment. (A) 400m walk time data across the female and male tertiles. (B) Titin N-fragment serum concentration data in the female and male tertiles. (C) Titin N-fragment serum concentration data in the young and old groups (PDF 234 KB). [file 11357_2024_1449_MOESM1_ESM.pdf]

S2A

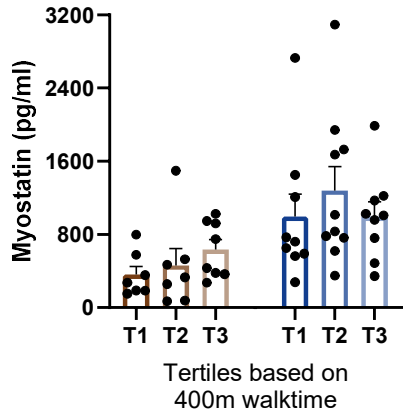

B

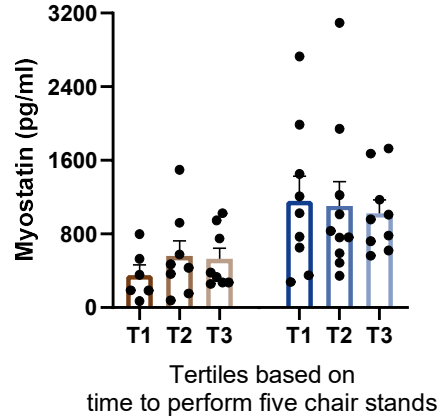

C

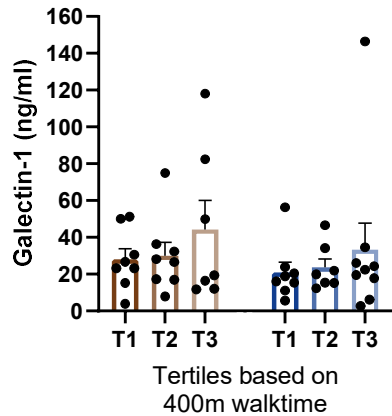

D

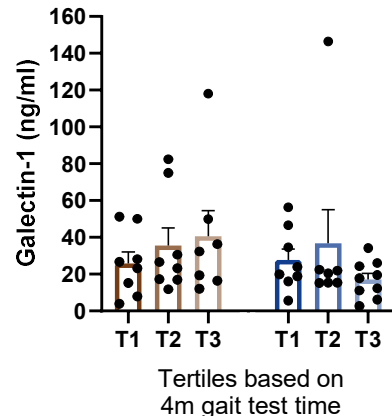

E

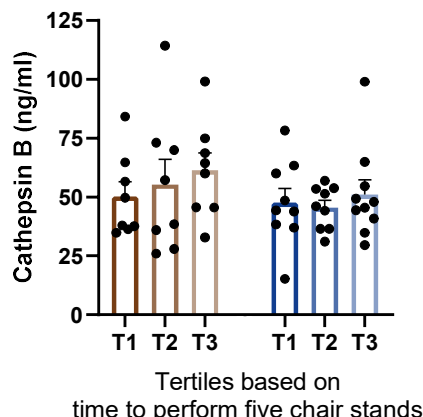

F

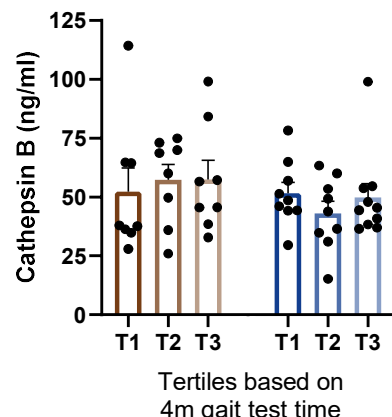

G

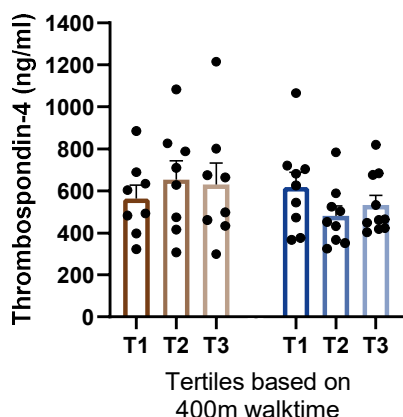

H

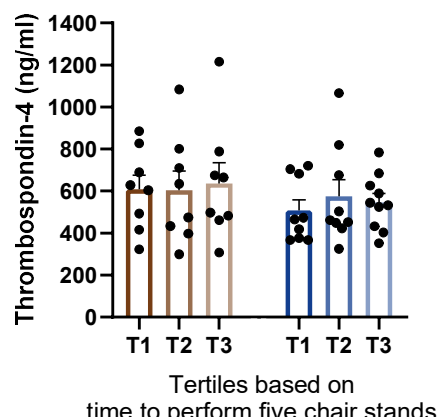

Supplement: Supplementary file 2 — Supplementary file2; Suppl. Fig. 2. Data of serum concentrations of Myostatin (A+B), Galectin-1 (C+D), Cathepsin B (E+F) and Thrombospondin-4 (G+H) using tertiles based on alternative physical function tests, i.e. the physical function tests these biomarkers did not correlate with based on the RNA-seq data (PDF 224 KB). [file 11357_2024_1449_MOESM2_ESM.pdf]
