# Supplementary material for: Blood-based biomarkers for early frailty are sex-specific: validation of a combined in silico prediction and data-driven approach
Source: GeroScience. 2024 Dec 3;47(3):3741–58. doi: 10.1007/s11357-024-01449-w (PMC12181598; doi:10.1007/s11357-024-01449-w)
Supplement: Supplementary file 3 — Supplementary file3; Suppl. Fig. 3. Correlations between biomarker serum concentrations for (A) females and (B) males. *p 0.05 (PDF 543 KB). [file 11357_2024_1449_MOESM3_ESM.pdf]

S3A

Females	Myostatin(total)_(pg/ml)	Galectin-1_(ng/ml)	Cathepsin_B_(ng/ml)	THBS4_(ng/ml)	Titin_(pmol/L)
**Myostatin(total)_(pg/ml)**	1.00	0.39	0.15	0.04	-0.30
**Galectin-1_(ng/ml)**	0.39	1.00	**0.45***	0.10	-0.13
**Cathepsin_B_(ng/ml)**	0.15	**0.45***	1.00	0.33	0.21
**THBS4_(ng/ml)**	0.04	0.10	0.33	1.00	0.27
**Titin_(pmol/L)**	-0.30	-0.13	0.21	0.27	1.00

B

Males	Myostatin(total)_(pg/ml)	Galectin-1_(ng/ml)	Cathepsin_B_(ng/ml)	THBS4_(ng/ml)	Titin_(pmol/L)
**Myostatin(total)_(pg/ml)**	1.00	**0.72***	0.07	0.04	0.10
**Galectin-1_(ng/ml)**	**0.72***	1.00	0.12	0.11	0.00
**Cathepsin_B_(ng/ml)**	0.07	0.12	1.00	0.22	0.13
**THBS4_(ng/ml)**	0.04	0.11	0.22	1.00	0.11
**Titin_(pmol/L)**	0.10	0.00	0.13	0.11	1.00
