# Supplementary material for: Blood-based biomarkers for early frailty are sex-specific: validation of a combined in silico prediction and data-driven approach
Source: GeroScience. 2024 Dec 3;47(3):3741–58. doi: 10.1007/s11357-024-01449-w (PMC12181598; doi:10.1007/s11357-024-01449-w)
Supplement: Supplementary file 5 — Supplementary file5; Suppl. Table 2. Quality control data of ELISA kits used in this study (DOCX 16 KB). [file 11357_2024_1449_MOESM5_ESM.docx]

|  | Company | Product number | Recovery spike test (%) | Serial dilution correlation (R) | Average intra-assay CV |
| --- | --- | --- | --- | --- | --- |
| Myostatin | R&D systems | DY788-05 | 71.2% | 0.998 | 1.3% |
| Galectin-1 | R&D systems | DY1152-05 | 89.9% | 0.999 | 6.1% |
| Cathepsin B | R&D systems | DY2176 | 98.1% | 0.999 | 2.1% |
| Thrombospondin-4 | Thermofisher Scientific | EH473RB | 110.2% | 0.996 | 4.2% |
| Titin N-fragment | IBL | 27902 | 81% | 0.994 | 4.4% |
